# Unexpected “Lazarus response” to single-agent bevacizumab in heavily pretreated patients with HER2-positive breast cancer

**DOI:** 10.37349/etat.2023.00189

**Published:** 2023-12-06

**Authors:** Alexey V. Emshanov, Denis V. Nesterov, Tatyana N. Sokolova, Priscilla S. Amankwah, Evgeny N. Imyanitov

**Affiliations:** Istituto Nazionale Tumori-IRCCS-Fondazione G. Pascale, Italy; ^1^Department of Cancer Therapy, Regional Oncology Hospital, Rostov-on-Don 344006, Russia; ^2^Department of Tumor Growth Biology, N.N. Petrov Institute of Oncology, Saint Petersburg 197758, Russia; ^3^Department of Medical Genetics, Saint Petersburg Pediatric Medical University, Saint Petersburg 194100, Russia; ^4^Laboratory of Molecular Genetics, Kurchatov Complex for Medical Primatology, National Research Centre “Kurchatov Institute”, Sochi 354376, Russia

**Keywords:** Bevacizumab, breast cancer, edema, HER2, single-agent therapy

## Abstract

Early clinical trials aimed to halt cancer progression by inhibiting the growth of new blood vessels in tumors through single-agent targeted therapy with bevacizumab. These trials largely proved unsuccessful. However, bevacizumab turned out to be efficient when administered in combination with other anticancer drugs. The efficacy of this approach is explained by the ability of bevacizumab to eliminate immature blood vessels thus normalizing intratumoral blood flow and improving the delivery of cytotoxic or targeted agents. This report describes four cases of heavily pretreated patients with metastatic HER2-positive breast cancer, who had no meaningful treatment options left, and who received single-agent bevacizumab as an empirical last-resort therapy. Three of these patients had severe complaints, and they demonstrated striking symptomatic relief within the first day of this treatment. In addition to the observed “Lazarus response”, which was likely attributed to the bevacizumab-driven resolution of edema, some evidence of a direct antitumor effect was observed. These data may call for the reconsideration of bevacizumab monotherapy in patients with HER2-associated breast cancer, and perhaps in some other categories of cancer patients.

## Introduction

Bevacizumab was developed as an antiangiogenic targeted drug. The initial hypothesis underlying the use of bevacizumab was that tumor viability is dependent on the formation of new blood vessels. Contrary to initial expectations, early studies with bevacizumab as monotherapy failed to yield significant clinical benefit [1–5]. However, this drug appeared to have substantial efficacy when used in combination with other anticancer agents [1, 6, 7]. These observations led to the revision of the initial hypothesis. It has now been proposed that the mechanism of action of bevacizumab is limited to the elimination of immature disorganized vasculature, which emerges during tumor development due to the unregulated expression of angiogenic factors by malignant cells. Bevacizumab is therefore believed to “normalize” intratumoral blood circulation. Consequently, this drug resolves edema, provides oxygen supply for cell division thereby increasing tumor sensitivity to cytotoxic agents, and improves drug delivery [2, 6, 7]. Bevacizumab is currently used as a single agent only in the treatment of glioblastomas, which are known to have abnormally high vascularization, while other approved indications allow the administration of this drug in combination with other anticancer compounds [6–8]. This report describes four cases, only in which intravenous bevacizumab infusions produced unexpected and pronounced beneficial effects when empirically provided to heavily pretreated, terminally ill HER2-positive breast cancer patients.

## Case report

**Figure 1:**
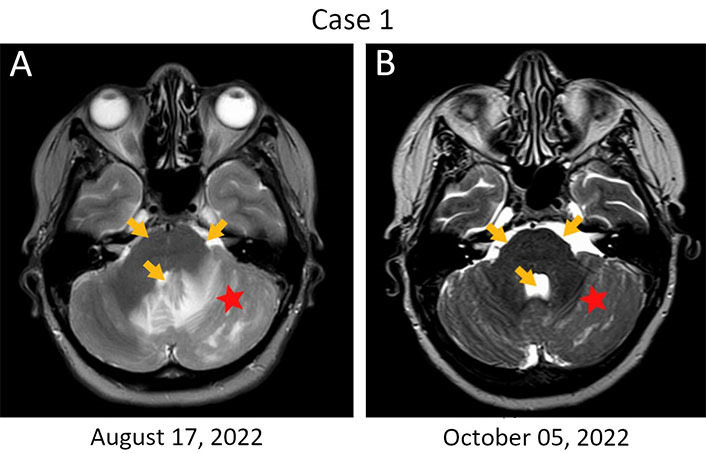
Rapid resolution of edema in a HER2-positive breast cancer patient with metastatic involvement of the brain. (A) Brain lesions at baseline. The first infusion of bevacizumab was given on September 6, 2022, when the patient was in coma; (B) MRI examination was performed on day 30 after the start of this treatment. Monotherapy with bevacizumab resulted in a significant reduction of the perifocal swelling in the left hemisphere of the cerebellum (red star); there is also a resolution of the compression of the pontine cistern and the fourth ventricle (orange arrows)

Patient 1, 54 years old, was diagnosed with estrogen receptor (ER^–^)/progesterone receptor (PR^‒^)/HER2^+++^ locally advanced (T4bN0M0) breast cancer in August 2019 (Table 1). She received neoadjuvant therapy [4 three-weekly cycles of doxorubicin (60 mg/m^2^) and cyclophosphamide (600 mg/m^2^) followed by 4 three-weekly cycles of docetaxel (75 mg/m^2^), trastuzumab (loading dose 8 mg/kg, then 6 mg/kg) and pertuzumab (loading dose 840 mg, then 420 mg)] [9]. The patient underwent surgery in March 2020, and the examination of excised tissues revealed a pathological complete response. The patient received standard adjuvant therapy consisting of 14 cycles of trastuzumab and pertuzumab from April to November 2020. A relapse occurred in September 2021 manifesting as a tumor lump (18 mm × 14 mm) in the cerebellum coupled with the metastatic involvement of the meninges. The possibility of radiological treatment was discussed, however, this option was not considered feasible due to extensive leptomeningeal involvement and large size of the cerebellar lesion. The patient received 6 three-weekly cycles of carboplatin [area under the curve (AUC) 5] and docetaxel (75 mg/m^2^) coupled with 10 cycles of trastuzumab (8 mg/kg, then 6 mg/kg) and pertuzumab (840 mg, then 420 mg), starting from October 2021. Enlargement of brain metastases was observed in April 2022, which warranted a switch to lapatinib (1,250 mg/day) and capecitabine (2,000 mg/m^2^ through days 1‒14 of a three-weekly cycle) [9]. This therapy yielded no benefit, and the patient went into a coma in August 2022. Standard antiedema therapy did not improve the patient’s condition, so the life expectancy for this woman did not exceed a few days. Recognizing that bevacizumab may decrease intratumoral interstitial pressure and alleviate metastasis-related cerebral edema [10, 11], 800 mg (10 mg/kg) of this drug was administered on September 6, 2022. Strikingly, the patient regained full consciousness within 24‒48 h, along with some mobility. The patient received a second 10 mg/kg dose of the drug after 2 weeks and then switched to three-weekly 15 mg/kg bevacizumab cycles. Magnetic resonance imaging (MRI) examination was carried out at the beginning of October and demonstrated the resolution of the edema (Figure 1). The patient remained stable for 3.5 months on bevacizumab monotherapy before the disease progression. Trastuzumab emtansine (T-DM1) was given at a dose of 3.6 mg/kg every three weeks, however, this treatment was unsuccessful and the woman died at the beginning of March 2023.

**Table 1:** Brief description of the treatment for the patients receiving bevacizumab monotherapy

**Patient**	**Age**	**IHC detection**	**Date of breast cancer diagnosis**	**Neoadjuvant therapy**	**Surgery**	**Duration of relapse-free interval, months**	**Date of the detection of metastatic disease**	**Conventional treatment of metastatic disease**	**Date of bevacizumab monotherapy administration**	**Main benefit from bevacizumab, its duration**	**Survival after the start of bevacizumab, months**
Patient 1	54 years	ER^–^/PR^–^/HER2^+++^	August 2019	Doxo + Cyclo, Tax + Trast + Pert	March 2020	19	September 2021	Carbo + Tax + Trast + Pert, Trast + Pert, Lapa + Cape	September 2022	Immediate resolution of coma, 3.5 months	6
Patient 2	68 years	ER^+^/PR^–^/HER2^+++^	April 2017	*	*	*	April 2017	Doxo + Cyclo, Tax, Carbo + Tax + Trast + Zole, Cape, anastrozole, Tax + Trast + Pert, Trast + Pert, Lapa + Trast, T-DM1	September 2022	Immediate resolution of neurological symptoms, 3.5 months	12+
Patient 3	52 years	ER^+^/PR^–^/HER2^+++^	December 2015	Cyclo + mitoxantrone + 5-FU	March 2016	27	June 2018	T-DM1, WBRT, Carbo + Tax + Trast + Pert, Trast + Pert, RS, Trast + Pert	December 2022	Partial response, 9+ months	9+
Patient 4	62 years	ER^–^/PR^–^/HER2^+++^	May 2011	None	May 2011	68	January 2017	Tax + Trast, Trast, Carbo + Cape + Trast, Lapa + Cape, T-DM1, Carbo + Tax + Trast + Pert, Trast + Pert, eribulin + Trast, Lapa + Cape, Lapa + Trast	September 2021	Immediate restoration of normal breathing, regression of superficial neck metastases; the duration of the effect could not be estimated because paclitaxel was added starting from the 2nd cycle	5 (death from sepsis)

* Not applicable: initially metastatic disease. IHC: immunohistochemistry; RS: radiosurgery; Doxo: doxorubicin; Cyclo: cyclophosphamide; Tax: taxanes; Trast: trastuzumab; Pert: pertzumab; Carbo: carboplatin; Lapa: labatinib; Cape: capecitabine; Zole: zoledronic acid; 5-FU: 5-fluorouracil; WBRT: whole-brain radiotherapy

**Figure 2:**
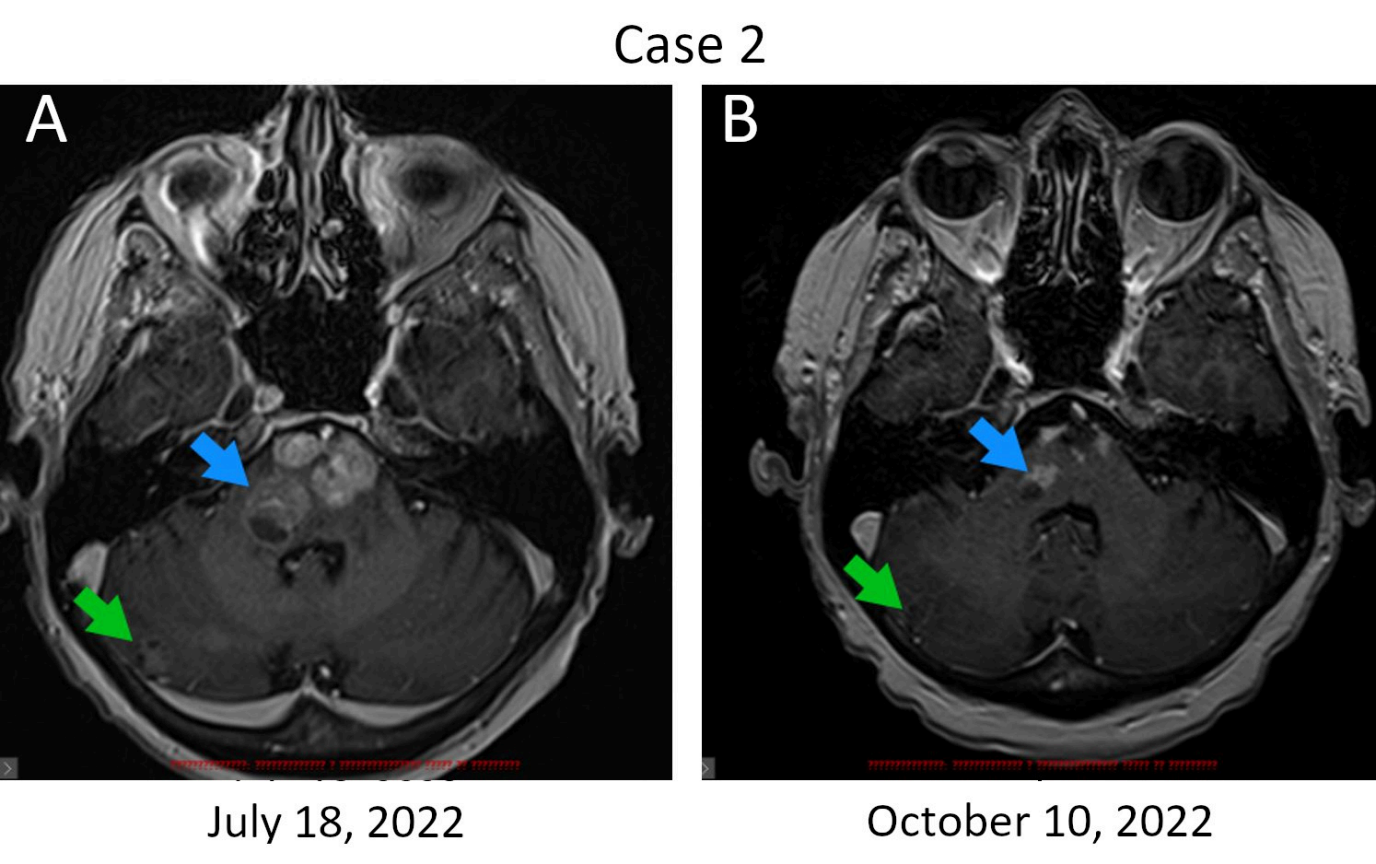
Regression of brain metastases in response to bevacizumab therapy. (A) Metastatic lesions in the brain at baseline. Bevacizumab infusions were started on September 16, 2023; (B) MRI examination was carried out on day 24 after the first dose of this drug. The reduction of the size of metastatic lumps is observed in the pons Varolii (from 19 mm × 15 mm to 13 mm × 8 mm and from 14 mm × 8 mm to 8 mm × 5 mm, blue arrow) and in the right hemisphere of the cerebellum (from 11 mm × 10 mm to 9 mm × 6 mm, green arrow). The sum of diameters of target lesions has been reduced by 32%

Patient 2, 68 years old, was diagnosed with ER^+^/PR^–^/HER2^+++^ metastatic (T1aN1M1) breast cancer in April 2017 (Table 1). Distant disease spread was manifested by multiple lesions in lungs and bones. She received 4 three-weekly cycles of doxorubicin (60 mg/m^2^) and cyclophosphamide (600 mg/m^2^) followed by 1 cycle of docetaxel (75 mg/m^2^) and was subsequently treated with paclitaxel (175 mg/m^2^), carboplatin (AUC 5), trastuzumab (8 mg/kg, then 6 mg/kg) and zoledronic acid (4 mg). Multiple supratentorial brain metastases and the involvement of pons Varolii were discovered in March 2021. The patient was given 6 cycles of capecitabine (2,000 mg/m^2^ through days 1‒14 every three weeks) followed by the administration of anastrozole (1 mg/day). Multiple new metastases in the brain, thoracic cavity, and bones were detected in September 2021. The patient received 6 cycles of docetaxel/trastuzumab/pertuzumab, followed by 3 cycles of trastuzumab/pertuzumab at standard doses [9]. This treatment did not stop the disease progression. A switch in medication was made to lapatinib (1,250 mg daily) and trastuzumab (6 mg/kg) in May 2022, and then to T-DM1 (3.6 mg/kg) in July 2022. The patient’s neurological state deteriorated significantly, so 400 mg (5 mg/kg) of bevacizumab was given on September 16, 2022. Remarkable symptomatic relief was observed within the first day after the infusion of this drug. She continued to receive bevacizumab [800 mg (10 mg/kg)] with two-week intervals for 2 cycles, and then switched to a dose of 15 mg/kg given every 3 weeks. MRI examination on October 10, 2022, revealed a reduction in the size of some brain metastases (Figure 2). Strictly speaking, it remains unclear whether this effect is attributed to T-DM1 or to bevacizumab. However, given the deteriorating condition of the patient while receiving the former and the rapid improvement observed upon administering the latter drug, it seems plausible to conclude that the benefit was derived from bevacizumab. Border-line enlargement of the lesions was noted on February 7, 2023. Docetaxel (75 mg/m^2^) and carboplatin (AUC 5) were added to bevacizumab at the end of April 2023, however, 3 cycles of this triplet therapy did not improve the condition of this woman. The patient is currently receiving symptomatic therapy.

**Figure 3:**
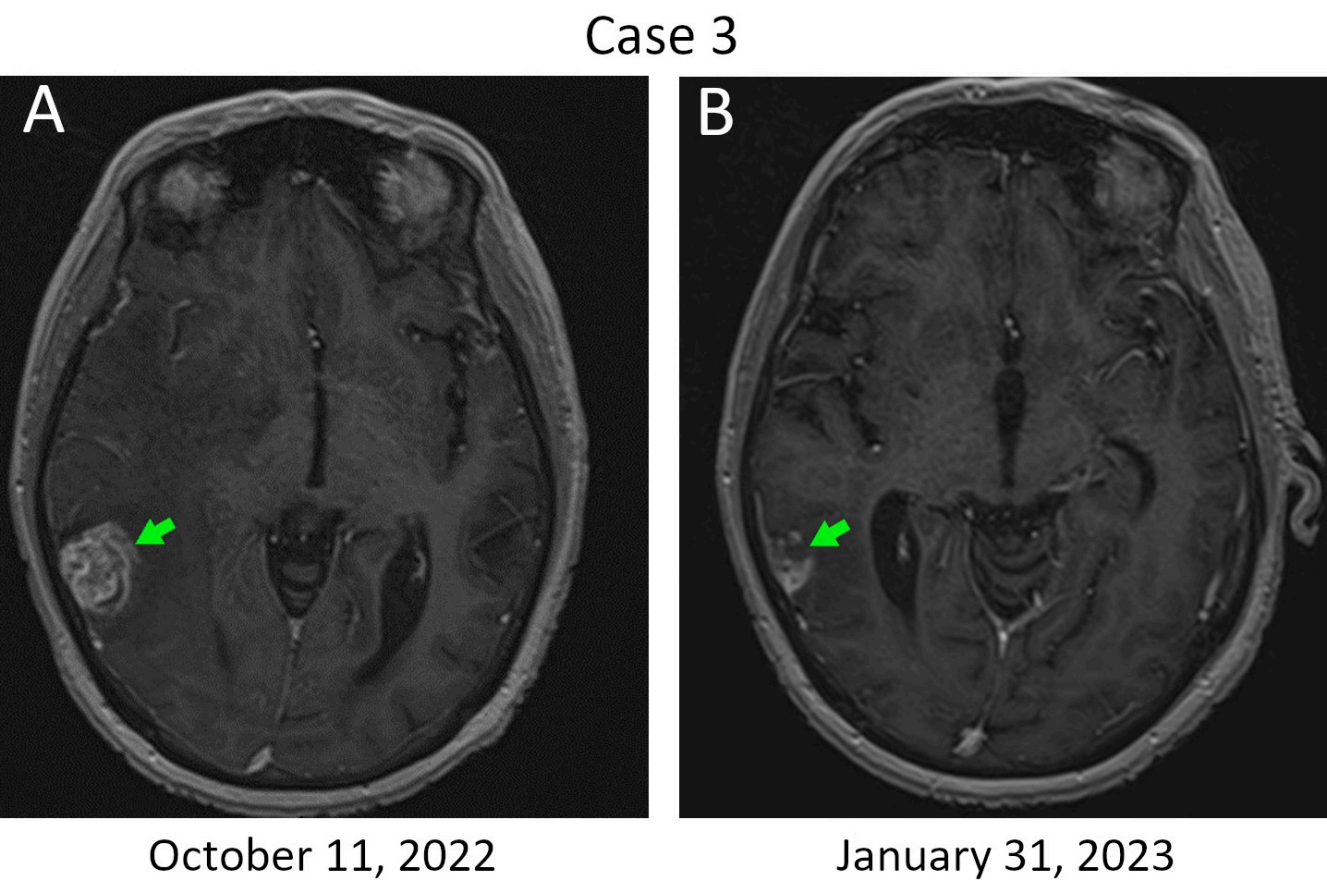
The response of metastatic lesions located in the right temporal lobe to a single-agent bevacizumab therapy. (A) Metastatic lump at baseline. Bevacizumab therapy was started on December 8, 2022; (B) MRI imaging was performed on day 54 after the initiation of this treatment. The baseline size of the metastasis is 24 mm × 21 mm (green arrow, left), while a 45% reduction in the tumor size was observed in the next examination (13 mm × 12 mm; green arrow, right)

Patient 3, 52 years old, was diagnosed with ER^+^/PR^–^/HER2^+++^ T4N3M0 breast cancer in the year 2015 (Table 1). She received 2 three-weekly cycles of neoadjuvant therapy consisting of cyclophosphamide (500 mg/m^2^), mitoxantrone (12 mg/m^2^), and 5-FU (500 mg/m^2^) and underwent radical surgery in March 2016. Morphological examination of the surgically excised tissues revealed invasive carcinoma in the breast, accompanied by metastatic involvement of axillary and subclavian lymph nodes. The patient received 6 cycles of conventional adjuvant taxane-based therapy combined with trastuzumab, radiation therapy, and then continued to receive a single-agent trastuzumab until May 2017. An extensive metastatic spread to the lymph nodes, bones, and liver was diagnosed in June 2018. The patient received 16 cycles of T-DM1 (3.6 mg/kg every three weeks). Multiple metastases in the brain were detected in September 2019, and the patient was subjected to whole brain irradiation. She then received 6 cycles of carboplatin/docetaxel/trastuzumab/pertuzumab, followed by 52 cycles of trastuzumab/pertuzumab at standard doses [9]. Stereotactic RS was performed for 8 brain metastases in December 2020 using the Novalis Tx equipment. Trastuzumab/pertuzumab therapy was discontinued in November 2022 due to disease progression in the brain, although the patient did not have neurological symptoms. Single-agent bevacizumab was given for 4 cycles every 2 weeks at a dose of 10 mg/kg, starting from December 8, 2022. An evident regression of brain metastatic lesions was detected by MRI on January 31, 2023 indicating a partial response by the response evaluation criteria in solid tumors (RECIST, Figure 3). Furthermore, the resolution of brain edema was observed (data not shown). The patient received stereotaxic treatment for brain metastases at the beginning of March 2023. Bevacizumab was resumed in the middle of March 2023 at a dose of 15 mg/kg, given every 3 weeks. Subsequent regular examinations did not reveal evidence of the disease progression. This patient currently remains in good condition, has returned to work, can perform normal daily activities, and continues to receive bevacizumab monotherapy.

Patient 4, 62 years old, was diagnosed with ER^–^/PR^–^/HER2^+++^ T2N0M0 breast cancer in the year 2011 (Table 1). Seven years after the surgery she developed massive metastatic involvement of the anterior thoracic wall and the neck, including axillary lymph nodes, submaxillary and parotid regions, and the salivary glands. Multiple lines of chemotherapy combined with HER2-targeted drugs failed to stop tumor growth. Being in terminal condition due to compression of the respiratory tract by metastatic lumps, the patient received a single dose of 800 mg bevacizumab (10 mg/kg) on September 20, 2021. The patient’s normal breathing was restored within 12‒24 h after the infusion of this drug. Surprisingly, regression of superficial neck metastases was detected within 2 weeks of bevacizumab therapy, followed by wound formation (data not shown). Weekly paclitaxel (90 mg/m^2^) was subsequently added to bevacizumab. However, the patient developed severe bacterial infections and died from sepsis in February 2022.

## Discussion

Medical literature sometimes uses the term “Lazarus response” for description of immediate and unexpected treatment effects. This wording refers to a biblical miracle in which Jesus visited Bethany and returned back to the life Lazarus, who died four days before and had already been entombed [12]. Rapid symptomatic effect was observed in three of the cases described in this report. The fourth patient did not have overt symptoms despite significant brain involvement. It is very likely that the striking improvement of the condition of these women was mainly attributed to the bevacizumab-driven resolution of edema, which corresponds well to preclinical data [2, 6] and clinical experience concerning the use of this drug [7, 10, 11]. In addition, a direct antitumor effect of bevacizumab was seen in some of the described cases (Figure 3). Bevacizumab is potentially compatible with the use of RS [13], therefore, clinical trials involving combined utilization of these treatments for eradication of brain metastases look feasible.

It is noteworthy that objective responses to bevacizumab monotherapy were observed in early breast cancer studies. Indeed, Cobleigh et al. [14] utilized this drug in 75 pretreated patients with metastatic disease and observed 5 confirmed and 2 unconfirmed instances of tumor size reduction. Single-agent bevacizumab also demonstrated reasonable efficacy in pancreatic neuroendocrine tumors: in the trial involving 24 patients, there were 3 subjects with partial response and 18 patients with stable disease for a period of at least 6 months [15]. Bevacizumab maintenance therapy has shown promise in a number of clinical studies [16]. This drug was also reported to delay the recurrence of surgically treated melanoma [17]. Taken together, these data may call for revisiting the role of single-agent bevacizumab in cancer management.

The present report was not designed as a prospective study but describes empirical clinical experience. It is limited to HER2-driven breast cancer, which is characterized by highly aggressive behavior and frequent brain involvement. The development of anti-HER2 targeted drugs led to dramatic improvement of life expectancy of women with this disease [18]. This report offers an additional treatment option for this category of patients. It remains to be investigated if the observed effects are applicable to other tumor types. If these data will be reproduced by independent researchers, it will be desirable to perform a more in-depth analysis of responding patients with regard to their baseline characteristics and changes occurring in the tumor tissues upon bevacizumab administration.

In conclusion, the described cases may call for reconsideration of bevacizumab monotherapy in patients with HER2-associated breast cancer and, perhaps, in some other categories of cancer patients. The efficacy of bevacizumab in subjects with tumor-related edema deserves particular appreciation.

## Abbreviations

5-FU: 5-fluorouracil
AUC: area under the curve
ER: estrogen receptor
MRI: magnetic resonance imaging
PR: progesterone receptor
RS: radiosurgery
T-DM1: trastuzumab emtansine

## Declarations

### Copyright

© The Author(s) 2023.
